# 3-(4-Bromo­phenyl­sulfon­yl)-5-chloro-2-methyl-1-benzofuran

**DOI:** 10.1107/S1600536812024592

**Published:** 2012-06-13

**Authors:** Hong Dae Choi, Pil Ja Seo, Uk Lee

**Affiliations:** a Department of Chemistry, Dongeui University, San 24 Kaya-dong, Busanjin-gu, Busan 614-714, Republic of Korea; b Department of Chemistry, Pukyong National University, 599-1 Daeyeon 3-dong, Nam-gu, Busan 608-737, Republic of Korea

## Abstract

In the title compound, C_15_H_10_BrClO_3_S, the 4-bromo-substituted benzene ring forms a dihedral angle of 72.55 (6)° with the mean plane [mean deviation = 0.008 (2) Å] of the benzofuran ring system. In the crystal, mol­ecules are linked by weak C—H⋯O hydrogen bonds into chains along [001]. There are also π–π inter­actions between the furan and benzene rings of symmetry-related benzofuran systems [centroid–centroid distances = 3.549 (3) and 3.632 (3) Å].

## Related literature

For background information and the crystal structures of related compounds, see: Choi *et al.* (2008 ▶, 2010 ▶).
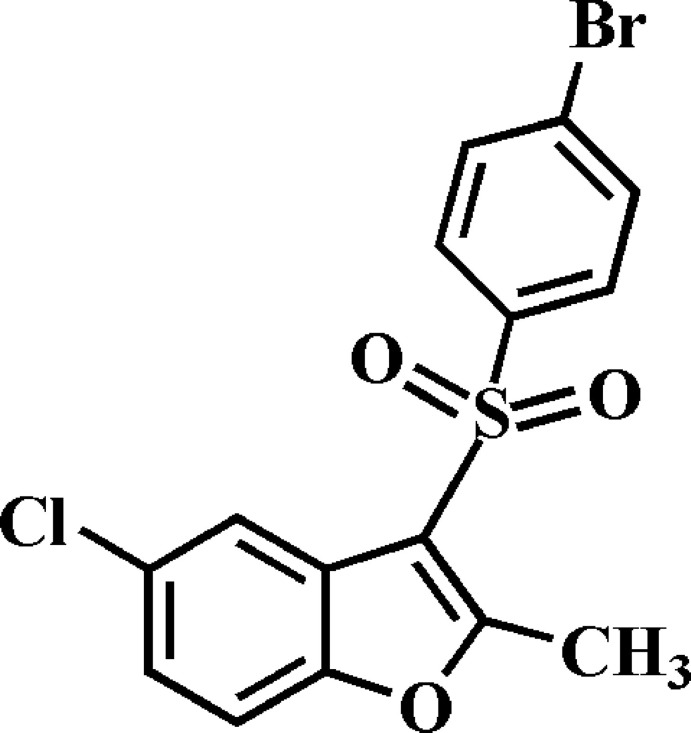


## Experimental

### 

#### Crystal data

C_15_H_10_BrClO_3_S
*M* *_r_* = 385.65
Triclinic, 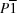

*a* = 7.1089 (2) Å
*b* = 10.4169 (3) Å
*c* = 11.0217 (3) Å
α = 69.659 (2)°
β = 89.526 (2)°
γ = 71.632 (1)°
*V* = 721.58 (4) Å^3^
*Z* = 2
Mo *K*α radiation
μ = 3.18 mm^−1^
*T* = 173 K
0.38 × 0.31 × 0.27 mm

#### Data collection

Bruker SMART APEXII CCD diffractometer
Absorption correction: multi-scan (*SADABS*; Bruker, 2009 ▶) *T*_min_ = 0.448, *T*_max_ = 0.746
13182 measured reflections
3570 independent reflections
3007 reflections with *I* > 2σ(*I*)
*R*_int_ = 0.054

#### Refinement

*R*[*F* ^2^ > 2σ(*F* ^2^)] = 0.036
*wR*(*F* ^2^) = 0.097
*S* = 1.08
3570 reflections
191 parameters
H-atom parameters constrained
Δρ_max_ = 0.45 e Å^−3^
Δρ_min_ = −0.43 e Å^−3^

### 

Data collection: *APEX2* (Bruker, 2009 ▶); cell refinement: *SAINT* (Bruker, 2009 ▶); data reduction: *SAINT*; program(s) used to solve structure: *SHELXS97* (Sheldrick, 2008 ▶); program(s) used to refine structure: *SHELXL97* (Sheldrick, 2008 ▶); molecular graphics: *ORTEP-3* (Farrugia, 1997 ▶) and *DIAMOND* (Brandenburg, 1998 ▶); software used to prepare material for publication: *SHELXL97*.

## Supplementary Material

Crystal structure: contains datablock(s) global, I. DOI: 10.1107/S1600536812024592/lh5482sup1.cif

Structure factors: contains datablock(s) I. DOI: 10.1107/S1600536812024592/lh5482Isup2.hkl

Supplementary material file. DOI: 10.1107/S1600536812024592/lh5482Isup3.cml

Additional supplementary materials:  crystallographic information; 3D view; checkCIF report

## Figures and Tables

**Table 1:** Hydrogen-bond geometry (Å, °)

*D*—H⋯*A*	*D*—H	H⋯*A*	*D*⋯*A*	*D*—H⋯*A*
C11—H11⋯O2^i^	0.95	2.53	3.203 (3)	128
C15—H15⋯O3^ii^	0.95	2.57	3.195 (3)	124

Symmetry codes: (i) 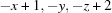; (ii) 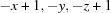.

**Fig. 1.**
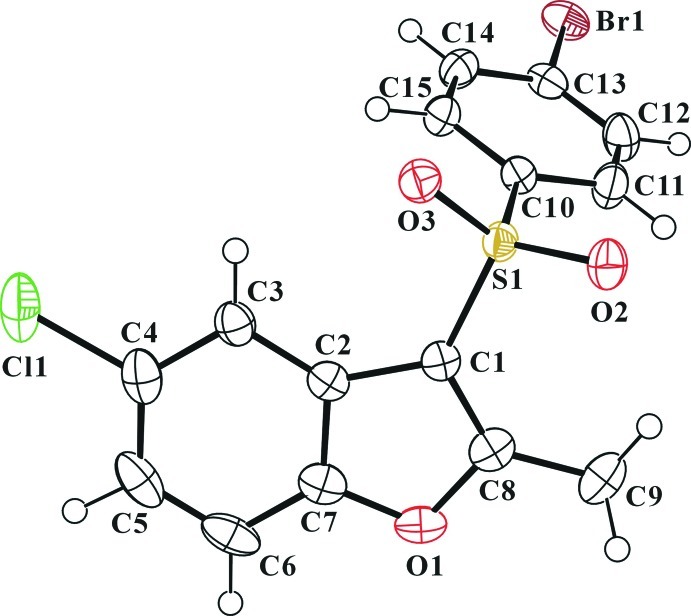
The molecular structure of the title compound with displacement ellipsoids drawn at the 50% probability level. H atoms are presented as small spheres of arbitrary radius.

**Fig. 2.**
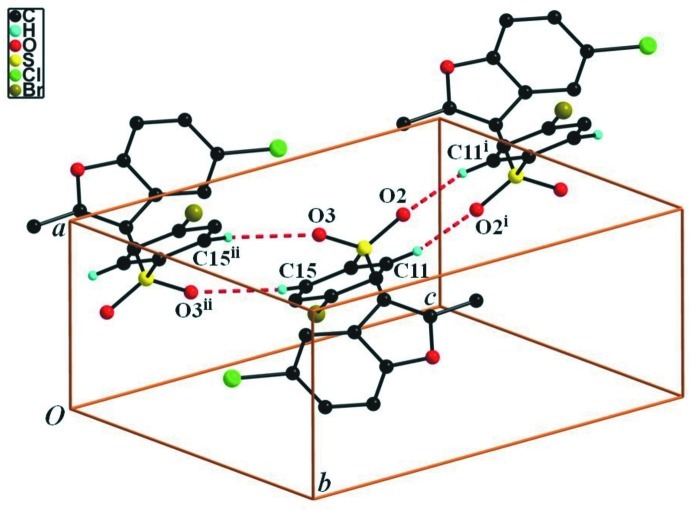
A view of the C—H···O interactions (dotted lines) in the packing of the title compound. H atoms non-participating in hydrogen-bonding are omitted for clarity. [Symmetry codes: (i) - x + 1, - y , - z + 2; (ii) - x + 1, - y , - z + 1].

**Fig. 3.**
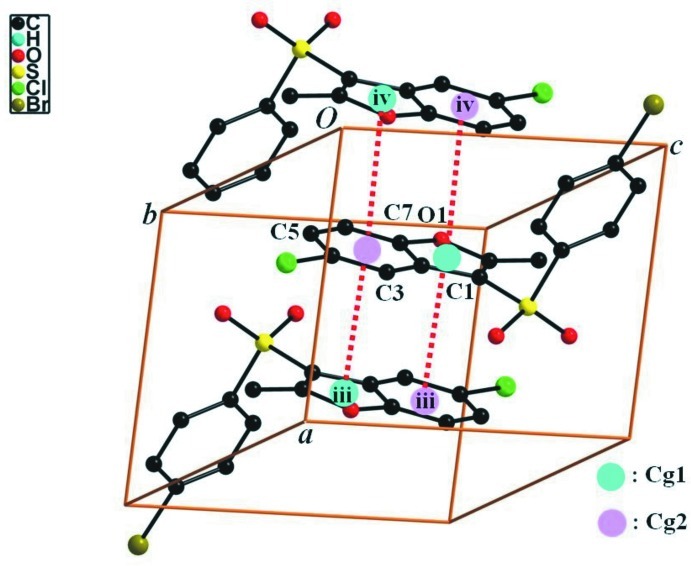
A view of the π–π interactions (dotted lines) in the crystal packing of the title compound. All H atoms are omitted for clarity. [Symmetry codes: (iii) - x + 1,- y + 1,- z + 1; (iv) - x, - y + 1,- z + 1].

## supplementary crystallographic information

### Comment

As a part of our ongoing study of 5-chloro-2-methyl-1-benzofuran derivatives containing 3-phenylsulfonyl (Choi *et al.*, 2008) and 3-(4-fluorophenylsulfonyl) (Choi *et al.*, 2010) substituents, we report herein the crystal structure of the title compound.

In the title molecule (Fig. 1), the benzofuran unit is essentially planar, with a mean deviation of 0.008 (2) Å from the least-squares plane defined by the nine constituent atoms. The dihedral angle between the 4-bromo substituted benzene ring and the mean plane of the benzofuran fragment is 72.55 (6)°. In the crystal structure (Fig. 2), molecules are connected by weak C—H···O hydrogen bonds (Table 1). The crystal packing (Fig. 3) also exhibits offset π–π interactions between the furan and benzene rings of neighbouring molecules, with Cg1···Cg2^iii^ & Cg1···Cg2^iv^ distances of 3.549 (3) Å & 3.632 (3) Å [Symmetry codes: (iii) - x + 1,- y + 1,- z + 1; (iv) - x, - y + 1,- z + 1] and interplanar distances of 3.510 (3) Å & 3.343 (3) Å resulting in slippages of 0.511 (3) Å & 1.420 (3) Å (Cg1 and Cg2 are the centroids of the C1/C2/C7/O1/C8 furan ring and the C2–C7 benzene ring, respectively).

### Experimental

3-Chloroperoxybenzoic acid (77%, 381 mg, 1.7 mmol) was added in small portions to a stirred solution of 3-(4-bromophenylsulfanyl)-5-chloro-2-methyl-1-benzofuran (318 mg, 0.8 mmol) in dichloromethane (40 mL) at 273 K. After being stirred at room temperature for 8h, the mixture was washed with saturated sodium bicarbonate solution and the organic layer was separated, dried over magnesium sulfate, filtered and concentrated at reduced pressure. The residue was purified by column chromatography (benzene) to afford the title compound as a colorless solid [yield 76%, m.p. 454-456 K; *R*_f_ = 0.71 (benzene)]. Single crystals suitable for X-ray diffraction were prepared by slow evaporation of a solution of the title compound in ethyl acetate at room temperature.

### Refinement

All H atoms were positioned geometrically and refined using a riding model, with C—H = 0.95 Å for aryl and 0.98 Å for methyl H atoms. U_iso_(H) = 1.2*U*_eq_(C) for aryl and 1.5*U*_eq_(C) for methyl H atoms. The positions of methyl hydrogens were optimized rotationally.

### Crystal data

**Table:**

C_15_H_10_BrClO_3_S	*Z* = 2
*M**_r_* = 385.65	*F*(000) = 384
Triclinic, *P*1	*D*_x_ = 1.775 Mg m^−^^3^
Hall symbol: -P 1	Mo *K*α radiation, λ = 0.71073 Å
*a* = 7.1089 (2) Å	Cell parameters from 7922 reflections
*b* = 10.4169 (3) Å	θ = 2.5–28.3°
*c* = 11.0217 (3) Å	µ = 3.18 mm^−^^1^
α = 69.659 (2)°	*T* = 173 K
β = 89.526 (2)°	Block, colourless
γ = 71.632 (1)°	0.38 × 0.31 × 0.27 mm
*V* = 721.58 (4) Å^3^	

### Data collection

**Table:**

Bruker SMART APEXII CCD diffractometer	3570 independent reflections
Radiation source: rotating anode	3007 reflections with *I* > 2σ(*I*)
Graphite multilayer monochromator	*R*_int_ = 0.054
Detector resolution: 10.0 pixels mm^-1^	θ_max_ = 28.3°, θ_min_ = 2.0°
φ and ω scans	*h* = −9→8
Absorption correction: multi-scan (*SADABS*; Bruker, 2009)	*k* = −13→13
*T*_min_ = 0.448, *T*_max_ = 0.746	*l* = −14→14
13182 measured reflections	

### Refinement

**Table:**

Refinement on *F*^2^	Primary atom site location: structure-invariant direct methods
Least-squares matrix: full	Secondary atom site location: difference Fourier map
*R*[*F*^2^ > 2σ(*F*^2^)] = 0.036	Hydrogen site location: difference Fourier map
*wR*(*F*^2^) = 0.097	H-atom parameters constrained
*S* = 1.08	*w* = 1/[σ^2^(*F*_o_^2^) + (0.0512*P*)^2^ + 0.0238*P*] where *P* = (*F*_o_^2^ + 2*F*_c_^2^)/3
3570 reflections	(Δ/σ)_max_ = 0.001
191 parameters	Δρ_max_ = 0.45 e Å^−^^3^
0 restraints	Δρ_min_ = −0.43 e Å^−^^3^

### Special details

**Table:**

Geometry. All esds (except the esd in the dihedral angle between two l.s. planes) are estimated using the full covariance matrix. The cell esds are taken into account individually in the estimation of esds in distances, angles and torsion angles; correlations between esds in cell parameters are only used when they are defined by crystal symmetry. An approximate (isotropic) treatment of cell esds is used for estimating esds involving l.s. planes.
Refinement. Refinement of F^2^ against ALL reflections. The weighted R-factor wR and goodness of fit S are based on F^2^, conventional R-factors R are based on F, with F set to zero for negative F^2^. The threshold expression of F^2^ > 2sigma(F^2^) is used only for calculating R-factors(gt) etc. and is not relevant to the choice of reflections for refinement. R-factors based on F^2^ are statistically about twice as large as those based on F, and R- factors based on ALL data will be even larger.

### Fractional atomic coordinates and isotropic or equivalent isotropic displacement parameters (Å^2^)

**Table:**

	*x*	*y*	*z*	*U*_iso_*/*U*_eq_	
Br1	−0.05797 (4)	−0.23812 (2)	0.82146 (3)	0.03829 (11)	
Cl1	0.31879 (13)	0.47313 (9)	0.12675 (7)	0.0514 (2)	
S1	0.53111 (8)	0.13394 (6)	0.70691 (5)	0.02350 (14)	
O1	0.1919 (2)	0.53895 (17)	0.62583 (18)	0.0320 (4)	
O2	0.6309 (3)	0.10707 (19)	0.83068 (17)	0.0320 (4)	
O3	0.6452 (2)	0.10389 (17)	0.60557 (16)	0.0282 (4)	
C1	0.3820 (3)	0.3152 (2)	0.6417 (2)	0.0237 (4)	
C2	0.3255 (3)	0.4018 (2)	0.5047 (2)	0.0242 (5)	
C3	0.3615 (4)	0.3785 (3)	0.3887 (2)	0.0288 (5)	
H3	0.4391	0.2863	0.3874	0.035*	
C4	0.2782 (4)	0.4967 (3)	0.2754 (3)	0.0345 (6)	
C5	0.1640 (4)	0.6333 (3)	0.2737 (3)	0.0406 (7)	
H5	0.1098	0.7108	0.1929	0.049*	
C6	0.1294 (4)	0.6564 (3)	0.3885 (3)	0.0378 (7)	
H6	0.0539	0.7491	0.3898	0.045*	
C7	0.2097 (4)	0.5387 (3)	0.5014 (3)	0.0293 (5)	
C8	0.2974 (4)	0.4014 (3)	0.7101 (3)	0.0297 (5)	
C9	0.2938 (4)	0.3784 (3)	0.8495 (3)	0.0400 (6)	
H9A	0.1613	0.3780	0.8744	0.060*	
H9B	0.3238	0.4568	0.8661	0.060*	
H9C	0.3940	0.2850	0.9008	0.060*	
C10	0.3644 (3)	0.0345 (2)	0.7367 (2)	0.0246 (5)	
C11	0.2993 (4)	−0.0064 (3)	0.8589 (2)	0.0333 (6)	
H11	0.3425	0.0202	0.9252	0.040*	
C12	0.1709 (4)	−0.0863 (3)	0.8835 (3)	0.0364 (6)	
H12	0.1249	−0.1146	0.9667	0.044*	
C13	0.1106 (4)	−0.1243 (2)	0.7867 (3)	0.0291 (5)	
C14	0.1743 (4)	−0.0834 (3)	0.6636 (2)	0.0305 (5)	
H14	0.1313	−0.1105	0.5976	0.037*	
C15	0.3008 (4)	−0.0028 (3)	0.6388 (2)	0.0286 (5)	
H15	0.3444	0.0272	0.5550	0.034*	

### Atomic displacement parameters (Å^2^)

**Table:**

	*U*^11^	*U*^22^	*U*^33^	*U*^12^	*U*^13^	*U*^23^
Br1	0.03630 (17)	0.02806 (14)	0.0493 (2)	−0.01477 (11)	0.00645 (13)	−0.00910 (12)
Cl1	0.0655 (5)	0.0720 (5)	0.0242 (3)	−0.0428 (4)	0.0035 (3)	−0.0084 (3)
S1	0.0238 (3)	0.0256 (3)	0.0201 (3)	−0.0069 (2)	0.0006 (2)	−0.0082 (2)
O1	0.0265 (9)	0.0271 (8)	0.0463 (11)	−0.0081 (7)	0.0063 (8)	−0.0187 (8)
O2	0.0309 (9)	0.0384 (9)	0.0253 (9)	−0.0116 (7)	−0.0032 (7)	−0.0097 (7)
O3	0.0263 (9)	0.0290 (8)	0.0267 (9)	−0.0060 (7)	0.0046 (7)	−0.0101 (7)
C1	0.0205 (11)	0.0259 (10)	0.0255 (12)	−0.0089 (9)	0.0020 (9)	−0.0092 (9)
C2	0.0192 (11)	0.0253 (10)	0.0294 (12)	−0.0104 (9)	0.0007 (9)	−0.0088 (9)
C3	0.0286 (12)	0.0300 (11)	0.0274 (13)	−0.0130 (10)	0.0027 (10)	−0.0071 (10)
C4	0.0331 (14)	0.0455 (14)	0.0267 (13)	−0.0246 (11)	0.0010 (11)	−0.0051 (11)
C5	0.0333 (14)	0.0357 (13)	0.0408 (17)	−0.0189 (11)	−0.0064 (12)	0.0071 (12)
C6	0.0244 (13)	0.0228 (11)	0.0604 (19)	−0.0103 (9)	−0.0014 (12)	−0.0059 (12)
C7	0.0219 (11)	0.0290 (11)	0.0397 (14)	−0.0129 (9)	0.0030 (10)	−0.0116 (10)
C8	0.0235 (12)	0.0351 (12)	0.0354 (14)	−0.0120 (10)	0.0042 (10)	−0.0165 (11)
C9	0.0392 (15)	0.0550 (16)	0.0365 (15)	−0.0181 (13)	0.0100 (12)	−0.0274 (13)
C10	0.0260 (12)	0.0228 (10)	0.0225 (11)	−0.0069 (9)	0.0015 (9)	−0.0061 (9)
C11	0.0420 (15)	0.0394 (13)	0.0227 (12)	−0.0190 (11)	0.0048 (11)	−0.0114 (11)
C12	0.0431 (15)	0.0384 (13)	0.0251 (13)	−0.0182 (12)	0.0050 (11)	−0.0042 (11)
C13	0.0275 (12)	0.0215 (10)	0.0334 (13)	−0.0065 (9)	0.0051 (10)	−0.0059 (9)
C14	0.0316 (13)	0.0337 (12)	0.0297 (13)	−0.0111 (10)	0.0020 (11)	−0.0153 (10)
C15	0.0312 (13)	0.0307 (11)	0.0242 (12)	−0.0097 (10)	0.0057 (10)	−0.0108 (10)

### Geometric parameters (Å, º)

**Table:**

Br1—C13	1.887 (2)	C6—C7	1.374 (4)
Cl1—C4	1.748 (3)	C6—H6	0.9500
S1—O2	1.4380 (18)	C8—C9	1.472 (4)
S1—O3	1.4392 (17)	C9—H9A	0.9800
S1—C1	1.735 (2)	C9—H9B	0.9800
S1—C10	1.765 (2)	C9—H9C	0.9800
O1—C8	1.374 (3)	C10—C11	1.387 (3)
O1—C7	1.378 (3)	C10—C15	1.389 (3)
C1—C8	1.364 (3)	C11—C12	1.385 (4)
C1—C2	1.446 (3)	C11—H11	0.9500
C2—C3	1.389 (3)	C12—C13	1.373 (4)
C2—C7	1.392 (3)	C12—H12	0.9500
C3—C4	1.383 (4)	C13—C14	1.393 (4)
C3—H3	0.9500	C14—C15	1.378 (4)
C4—C5	1.392 (4)	C14—H14	0.9500
C5—C6	1.375 (4)	C15—H15	0.9500
C5—H5	0.9500		
			
O2—S1—O3	120.14 (11)	C1—C8—O1	110.0 (2)
O2—S1—C1	108.61 (11)	C1—C8—C9	134.5 (2)
O3—S1—C1	106.53 (10)	O1—C8—C9	115.6 (2)
O2—S1—C10	107.53 (11)	C8—C9—H9A	109.5
O3—S1—C10	107.70 (10)	C8—C9—H9B	109.5
C1—S1—C10	105.43 (11)	H9A—C9—H9B	109.5
C8—O1—C7	107.14 (17)	C8—C9—H9C	109.5
C8—C1—C2	107.8 (2)	H9A—C9—H9C	109.5
C8—C1—S1	126.30 (19)	H9B—C9—H9C	109.5
C2—C1—S1	125.94 (17)	C11—C10—C15	120.7 (2)
C3—C2—C7	119.6 (2)	C11—C10—S1	119.19 (19)
C3—C2—C1	135.8 (2)	C15—C10—S1	120.08 (18)
C7—C2—C1	104.6 (2)	C12—C11—C10	119.5 (2)
C4—C3—C2	116.4 (2)	C12—C11—H11	120.3
C4—C3—H3	121.8	C10—C11—H11	120.3
C2—C3—H3	121.8	C13—C12—C11	119.5 (2)
C3—C4—C5	123.4 (3)	C13—C12—H12	120.2
C3—C4—Cl1	118.2 (2)	C11—C12—H12	120.2
C5—C4—Cl1	118.4 (2)	C12—C13—C14	121.5 (2)
C6—C5—C4	120.2 (2)	C12—C13—Br1	119.62 (19)
C6—C5—H5	119.9	C14—C13—Br1	118.8 (2)
C4—C5—H5	119.9	C15—C14—C13	118.9 (2)
C7—C6—C5	116.6 (2)	C15—C14—H14	120.5
C7—C6—H6	121.7	C13—C14—H14	120.5
C5—C6—H6	121.7	C14—C15—C10	119.8 (2)
C6—C7—O1	125.6 (2)	C14—C15—H15	120.1
C6—C7—C2	123.9 (2)	C10—C15—H15	120.1
O1—C7—C2	110.5 (2)		
			
O2—S1—C1—C8	−27.8 (2)	C1—C2—C7—O1	−0.6 (2)
O3—S1—C1—C8	−158.5 (2)	C2—C1—C8—O1	−0.8 (3)
C10—S1—C1—C8	87.2 (2)	S1—C1—C8—O1	179.12 (16)
O2—S1—C1—C2	152.06 (19)	C2—C1—C8—C9	178.4 (3)
O3—S1—C1—C2	21.3 (2)	S1—C1—C8—C9	−1.7 (4)
C10—S1—C1—C2	−92.9 (2)	C7—O1—C8—C1	0.4 (2)
C8—C1—C2—C3	−179.8 (3)	C7—O1—C8—C9	−179.0 (2)
S1—C1—C2—C3	0.3 (4)	O2—S1—C10—C11	22.3 (2)
C8—C1—C2—C7	0.8 (2)	O3—S1—C10—C11	153.12 (19)
S1—C1—C2—C7	−179.05 (17)	C1—S1—C10—C11	−93.4 (2)
C7—C2—C3—C4	0.4 (3)	O2—S1—C10—C15	−157.24 (19)
C1—C2—C3—C4	−178.8 (2)	O3—S1—C10—C15	−26.4 (2)
C2—C3—C4—C5	0.1 (4)	C1—S1—C10—C15	87.0 (2)
C2—C3—C4—Cl1	−179.96 (17)	C15—C10—C11—C12	0.5 (4)
C3—C4—C5—C6	0.3 (4)	S1—C10—C11—C12	−179.0 (2)
Cl1—C4—C5—C6	−179.7 (2)	C10—C11—C12—C13	0.3 (4)
C4—C5—C6—C7	−1.1 (4)	C11—C12—C13—C14	−0.5 (4)
C5—C6—C7—O1	−179.8 (2)	C11—C12—C13—Br1	178.11 (19)
C5—C6—C7—C2	1.7 (4)	C12—C13—C14—C15	0.0 (4)
C8—O1—C7—C6	−178.5 (2)	Br1—C13—C14—C15	−178.66 (18)
C8—O1—C7—C2	0.2 (2)	C13—C14—C15—C10	0.8 (4)
C3—C2—C7—C6	−1.4 (4)	C11—C10—C15—C14	−1.1 (4)
C1—C2—C7—C6	178.1 (2)	S1—C10—C15—C14	178.47 (18)
C3—C2—C7—O1	179.9 (2)		

### Hydrogen-bond geometry (Å, º)

**Table:**

*D*—H···*A*	*D*—H	H···*A*	*D*···*A*	*D*—H···*A*
C11—H11···O2^i^	0.95	2.53	3.203 (3)	128
C15—H15···O3^ii^	0.95	2.57	3.195 (3)	124

Symmetry codes: (i) −*x*+1, −*y*, −*z*+2; (ii) −*x*+1, −*y*, −*z*+1.
